# Supplementary material for: Markers of Oxidative Stress, Inflammation and Endothelial Function following High-Dose Intravenous Iron in Patients with Non-Dialysis-Dependent Chronic Kidney Disease—A Pooled Analysis
Source: Int J Mol Sci. 2022 Dec 16;23(24):16016. doi: 10.3390/ijms232416016 (PMC9787941; doi:10.3390/ijms232416016)
Supplement: Supplementary file 1 [file ijms-2083032-supplementary.pdf › ijms-2083032-supplementary.pdf]

Table S1: Pooled analysis data

Total population (n=36)					CKD stage 3b					CKD stage 4				
Variable	Mean	Median	SD	IQR	Variable	Mean	Median	SD	IQR	Variable	Mean	Median	SD	IQR
**Baseline - creatinine / µmol/L**	185.78	172.00	57.43	71.75	**Baseline - creatinine / µmol/L**	149.50	147.50	18.28	22.00	**Baseline - creatinine / µmol/L**	234.75	229.50	50.57	47.00
**1 month - creatinine / µmol/L**	187.90	173.00	69.89	72.50	**1 month - creatinine / µmol/L**	143.54	141.00	17.17	16.00	**1 month - creatinine / µmol/L**	238.27	214.00	69.74	47.00
**3 months - creatinine / µmol/L**	196.76	187.00	68.54	85.00	**3 months - creatinine / µmol/L**	148.64	148.00	22.33	20.00	**3 months - creatinine / µmol/L**	245.73	231.00	60.10	64.50
**Baseline - eGFR / mL/min per 1.73 m2**	32.77	32.63	10.87	18.58	**Baseline - eGFR / mL/min per 1.73 m2**	39.54	41.33	4.65	7.82	**Baseline - eGFR / mL/min per 1.73 m2**	22.17	23.00	3.97	5.22
**1 month - eGFR / mL/min per 1.73 m2**	33.10	33.88	11.78	17.86	**1 month - eGFR / mL/min per 1.73 m2**	41.44	38.88	6.65	10.29	**1 month - eGFR / mL/min per 1.73 m2**	23.05	24.00	5.92	6.81
**3 months - eGFR / mL/min per 1.73 m2**	31.25	27.79	11.64	19.11	**3 months - eGFR / mL/min per 1.73 m2**	39.72	38.97	6.96	7.84	**3 months - eGFR / mL/min per 1.73 m2**	21.88	22.00	4.99	7.43
**Baseline - Hemoglobin / g/L**	127.67	130.50	11.24	10.25	**Baseline - Hemoglobin / g/L**	131.81	131.00	8.08	5.50	**Baseline - Hemoglobin / g/L**	122.00	125.00	12.70	12.50
**1 month - Hemoglobin / g/L**	127.94	130.00	12.82	10.25	**1 month - Hemoglobin / g/L**	134.38	134.00	6.50	8.00	**1 month - Hemoglobin / g/L**	120.47	126.00	13.87	15.00
**3 months - Hemoglobin / g/L**	129.33	133.00	15.17	9.00	**3 months - Hemoglobin / g/L**	138.00	134.50	10.31	13.00	**3 months - Hemoglobin / g/L**	121.86	128.00	15.74	23.50
**Baseline - Serum ferritin / µg/l**	65.58	55.00	38.57	36.50	**Baseline - Serum ferritin / µg/l**	63.75	62.00	33.36	31.75	**Baseline - Serum ferritin / µg/l**	70.50	55.00	47.26	46.75
**1 month - Serum ferritin / µg/l**	279.58	257.00	124.65	111.00	**1 month - Serum ferritin / µg/l**	235.54	207.00	106.27	131.00	**1 month - Serum ferritin / µg/l**	311.40	294.00	137.94	161.50
**3 months - Serum ferritin / µg/l**	267.41	224.00	160.21	121.00	**3 months - Serum ferritin / µg/l**	204.55	218.00	75.98	74.00	**3 months - Serum ferritin / µg/l**	309.13	272.00	201.79	226.00
**Baseline - TSAT / %**	21.19	19.00	7.90	9.25	**Baseline - TSAT / %**	22.50	21.50	8.45	9.50	**Baseline - TSAT / %**	17.44	17.00	3.61	5.00
**1 month - TSAT / %**	27.53	27.50	10.44	14.25	**1 month - TSAT / %**	32.17	32.50	9.40	8.25	**1 month - TSAT / %**	21.87	21.00	8.72	8.50
**3 months - TSAT / %**	28.17	26.00	17.07	17.00	**3 months - TSAT / %**	27.73	30.00	11.00	16.00	**3 months - TSAT / %**	27.40	24.00	21.73	13.00
**Baseline - CRP / mg/L**	6.52	4.00	6.37	6.80	**Baseline - CRP / mg/L**	4.28	2.80	4.24	1.85	**Baseline - CRP / mg/L**	8.54	4.85	7.96	10.43
**1 month - CRP / mg/L**	5.85	4.00	7.05	3.70	**1 month - CRP / mg/L**	3.25	2.60	1.84	2.10	**1 month - CRP / mg/L**	8.14	4.80	9.51	5.90
**3 months - CRP / mg/L**	9.21	4.60	13.19	3.75	**3 months - CRP / mg/L**	9.78	5.45	8.97	12.15	**3 months - CRP / mg/L**	9.67	4.60	16.70	2.75
**Baseline - IL6 / pg/ml**	18.50	8.03	31.23	2.04	**Baseline - IL6 / pg/ml**	29.88	8.06	45.38	14.14	**Baseline - IL6 / pg/ml**	9.31	7.66	6.10	1.08
**1 month - IL6 / pg/ml**	22.02	7.92	34.85	2.36	**1 month - IL6 / pg/ml**	39.24	8.05	51.86	51.34	**1 month - IL6 / pg/ml**	11.09	7.92	9.82	1.28
**3 months - IL6 / pg/ml**	22.35	8.00	36.86	3.01	**3 months - IL6 / pg/ml**	37.48	8.16	51.38	49.61	**3 months - IL6 / pg/ml**	9.29	7.20	5.77	2.58
**Baseline - IL10 / pg/ml**	52.96	15.26	75.24	31.48	**Baseline - IL10 / pg/ml**	49.17	13.51	70.66	38.92	**Baseline - IL10 / pg/ml**	54.46	15.26	93.98	25.64
**1 month - IL10 / pg/ml**	52.79	17.41	74.43	31.86	**1 month - IL10 / pg/ml**	54.38	14.08	82.61	31.86	**1 month - IL10 / pg/ml**	46.55	22.24	73.87	20.75
**3 months - IL10 / pg/ml**	51.04	13.98	75.58	32.26	**3 months - IL10 / pg/ml**	50.51	14.55	76.59	35.00	**3 months - IL10 / pg/ml**	43.59	12.38	79.43	9.68
**Baseline - E-selectin / ng/ml**	56.74	54.86	24.27	29.76	**Baseline - E-selectin / ng/ml**	50.09	50.49	18.16	31.22	**Baseline - E-selectin / ng/ml**	65.43	62.13	31.99	37.23
**1 month - E-selectin / ng/ml**	62.07	58.87	25.35	32.50	**1 month - E-selectin / ng/ml**	55.34	52.06	17.92	28.91	**1 month - E-selectin / ng/ml**	67.69	60.08	32.55	41.96
**3 months - E-selectin / ng/ml**	60.37	61.16	20.19	37.20	**3 months - E-selectin / ng/ml**	54.92	52.62	19.77	33.36	**3 months - E-selectin / ng/ml**	65.91	66.08	22.17	34.84
**Baseline - P-selectin / ng/ml**	69.26	57.50	49.67	29.19	**Baseline - P-selectin / ng/ml**	58.99	49.00	30.86	24.22	**Baseline - P-selectin / ng/ml**	66.38	65.25	16.64	23.19
**1 month - P-selectin / ng/ml**	72.31	64.94	55.07	30.47	**1 month - P-selectin / ng/ml**	62.68	62.88	20.91	14.13	**1 month - P-selectin / ng/ml**	66.96	68.19	25.67	28.47
**3 months - P-selectin / ng/ml**	77.01	61.75	66.79	38.75	**3 months - P-selectin / ng/ml**	69.68	57.44	46.94	19.16	**3 months - P-selectin / ng/ml**	62.80	63.50	28.27	35.31
**Baseline - PWV / m/sec**	7.90	7.55	2.98	2.15	**Baseline - PWV / m/sec**	8.31	7.70	3.29	1.80	**Baseline - PWV / m/sec**	7.95	7.65	2.87	2.10
**1 month - PWV / m/sec**	7.89	7.60	2.14	2.60	**1 month - PWV / m/sec**	7.79	7.00	2.37	2.53	**1 month - PWV / m/sec**	7.88	7.90	2.07	1.80
**3 months - PWV / m/sec**	7.82	7.60	2.08	2.40	**3 months - PWV / m/sec**	7.68	7.60	2.19	2.70	**3 months - PWV / m/sec**	7.95	7.90	2.21	1.40
**Baseline - Aug Ind / %**	23.97	25.00	12.10	15.00	**Baseline - Aug Ind / %**	23.56	25.00	11.39	17.00	**Baseline - Aug Ind / %**	23.50	24.50	11.14	15.25
**1 month - Aug Ind / %**	19.97	20.00	10.82	17.00	**1 month - Aug Ind / %**	18.42	17.00	10.55	18.00	**1 month - Aug Ind / %**	19.62	20.00	7.91	12.00
**3 months - Aug Ind / %**	22.69	22.00	11.76	14.00	**3 months - Aug Ind / %**	22.92	19.00	14.00	14.00	**3 months - Aug Ind / %**	23.54	27.00	9.81	9.00
**Baseline - TBARS / nM**	939.64	958.21	321.49	304.45	**Baseline - TBARS / nM**	904.34	938.05	193.03	277.40	**Baseline - TBARS / nM**	876.53	960.49	291.34	301.99
**1 month - TBARS / nM**	966.29	1006.90	337.89	397.65	**1 month - TBARS / nM**	962.79	885.01	369.21	401.73	**1 month - TBARS / nM**	936.52	1039.02	329.83	377.99
**3 months - TBARS / nM**	985.26	1003.22	289.50	404.55	**3 months - TBARS / nM**	995.52	895.44	356.37	452.94	**3 months - TBARS / nM**	974.38	1019.63	207.07	399.99

Table S2: Statistical analysis as per variable of interest					
**Variable**	**Population**	**P-value**	**Variable**	**Population**	**P-value**
TBARS / nM	Total population	0.59	***E-selectin / ng/ml***	Total population	0.53
	CKD stage 3b	0.45		CKD stage 3b	0.47
	CKD stage 4	0.43		CKD stage 4	0.96
CRP / mg/L	Total population	0.27	***P-selectin / ng/ml***	Total population	0.64
	CKD stage 3b	0.03		CKD stage 3b	0.47
	CKD stage 4	0.79		CKD stage 4	0.73
IL6 / pg/ml	Total population	0.74	***PWV / m/sec***	Total population	0.90
	CKD stage 3b	0.76		CKD stage 3b	0.53
	CKD stage 4	0.95		CKD stage 4	1.00
IL10 / pg/ml	Total population	0.93	***Aug Ind / %***	Total population	0.60
	CKD stage 3b	0.97		CKD stage 3b	0.84
	CKD stage 4	0.80		CKD stage 4	0.95
